# Cystic fibrosis management in pediatric population—from clinical features to personalized therapy

**DOI:** 10.3389/fped.2024.1393193

**Published:** 2024-05-10

**Authors:** Alice Nicoleta Azoicai, Ancuta Lupu, Laura Mihaela Trandafir, Monica Mihaela Alexoae, Mirabela Alecsa, Iuliana Magdalena Starcea, Magdalena Cuciureanu, Anton Knieling, Delia Lidia Salaru, Elena Hanganu, Adriana Mocanu, Vasile Valeriu Lupu, Ileana Ioniuc

**Affiliations:** ^1^ Pediatrics, “Grigore T. Popa” University of Medicine and Pharmacy, Iasi, Romania; ^2^ Faculty of Medicine, “Grigore T. Popa” University of Medicine and Pharmacy, Iasi, Romania; ^3^ Department of Biomedical Sciences, “Grigore T. Popa” University of Medicine and Pharmacy, Iasi, Romania

**Keywords:** cystic fibrosis, children, screening, mutation, treatment

## Abstract

Cystic fibrosis (CF) is an autosomal recessive disease caused by mutations of the gene encoding the cystic fibrosis transmembrane conductance regulator (CFTR). In 1949, it's been identified as a monogenic disease and was thought to primarily affect individuals of Northern European descent. It was the most prevalent autosomal recessive disease that shortens life. With the availability of multiple testing methodologies nowadays, there is a chance to create novel and enhanced treatment options. Even in the absence of a high sweat chloride test (SCT) result, the discovery of two causal mutations is diagnostic for cystic fibrosis (CF). For a CF diagnosis, however, at least two positive E sweat chloride tests are still required. In order to achieve early and active intervention to manage cystic fibrosis (CF) and its comorbidities, treatment regimens for pediatric patients should be evaluated, improved, and closely monitored. New developments in the treatment of cystic fibrosis (CF) have led to the development of medications derived from molecules that target the pathogenetic pathway of the illness. These options are very efficient and allow pediatric patients to receive individualized care. However, in order to better direct patient care and enhance patient outcomes, it is crucial to research uncommon CF mutations, which can provide crucial information about the prognosis of the disease and the relationships between genotype and phenotype. To ensure the success of creating novel, safer, and more efficient treatment approaches, a deeper understanding of the pathogeny of the illness is required. In the age of customized medicine, genetic research will be essential to improving patient care and quality of life for those with uncommon mutations.

## 1 Introduction

Cystic fibrosis (CF) is an autosomal recessive disease caused by mutations of the gene encoding the cystic fibrosis transmembrane conductance regulator (CFTR) (1). It was first reported to the medical community in 1949, as a monogenic disease with autosomal recessive penetrance, affecting people of Northern European descent (1).

In the era of genetic testing, almost 1,000 cases per year of CF are newly diagnosed globally, with over 75% of CF patients tested under 2 years of age. Incidence rates vary widely around the world, but rates as high as 1 in 2,000–3,000 live births are associated with Caucasian populations with Northern European ancestry (2).

Clinical features include viscous secretions in the lungs, pancreas, liver, intestine, and reproductive tract and increased salt content in sweat gland products, the main cause of CF complications and patient mortality being progressive lung disease (2, 3). There are several hypotheses linking loss of channel function to lung pathogenesis, including airway surface dehydration, abnormal mucus properties and tethering to sub-mucosal glands. These initial events are supposed to lead to impaired mucus clearance and airway obstruction, making the lungs more vulnerable to infection, inflammation, and eventual structural damage (3). Therapeutic development focused on reversing the progressive obstructive lung disease can be mostly effective, as pulmonary manifestations are the major cause of morbidity and mortality in CF. There is variation regarding the course of the disease, beginning from a few months after birth to decades until the diagnostic is established. Many patients are exhibiting mild or atypical symptoms (4) therefore, clinicians should be able to rule out CF as a possible diagnosis in patients with few typical CF signs and symptoms.

Cystic fibrosis is caused by pathogenic mutations in a single large gene located on human chromosome 7 which encodes the cystic fibrosis transmembrane conductance regulator (CFTR) protein. CFTR is a member of ATP-binding cassette- ABC family of proteins, a large group of related proteins that share transmembrane transport functions (5). It forms a cell membrane-spanning chloride channel whose function is regulated by phosphorylation, mediated by cAMP-dependent phosphokinases (5). Thus, CFTR gene mutations result in production of defective proteins that cannot be processed normally by the endoplasmic reticulum, with defective transport to the cell membrane (5). Mutated CFTR protein molecules that can reach the cell membrane are dysfunctional and they cannot carry chloride outside the cell, leading to in-cell storage of chloride ions, water molecules in the epithelial cells and lack of hydration of extracellular mucus and secretions.

Since the molecular diagnosis ushered in a new era of available treatments over time six categories of CFTR mutations have been identified (6). F508del (with deletion of phenylalanine at site 508 caused by chromosomal deletion of three nucleotides, denoted c.1521_1523delCTT), a deletion causing protein misfolding, is the most prevalent pathogenic mutation discovered in Caucasians. The typical Class II mutation (mutations that cause early degradation or incomplete development) is Phe508del. Premature termination codons (PTCs) cause an incomplete synthesis, which is the hallmark of class I mutations (6). The Gly551Asp mutation (formerly known as G551D), which results in disordered and inhibited regulation and gating and reduced ATP binding and hydrolysis, is the class III mutation. Class IV mutations are confirmed by a deficient chloride conductance, which include the R117H mutation, also known as the Arg117His mutation. Class VI mutations, those that display reduced cell surface half-life (a characteristic shared by many Class II allele), as well as Class V mutations—those that exhibit decreased protein synthesis—are also described in the CF pathogenic pathway (5, 6).

## 2 Prenatal diagnostic of CF

Since CF is the most common autosomal recessive genetic disorder among Caucasians, the majority of carrier couples do not have a family history of the condition or any prior knowledge regarding the possibility of transmission. In order to identify early symptoms of the disease and receive an early diagnosis, parents, patients, relatives, and caregivers need extensive education (7). Prenatally, a hyperechoic bowel on an ultrasound can be an important issue regarding the possibility of CF. This hyperechoic bowel characteristic has been noted in 50%–78% of fetuses with known CF; in contrast, identifying hyperechoic bowel in a fetus without known CF is linked to a 0.8%–13.3% chance of a positive diagnosis (8). The percentage also depends on the parents’ ethnic backgrounds as well as other genetic and environmental factors because some diagnoses call for additional testing. Fetal hyperechoic bowel can be brought on by changed meconium consistency in the small intestine, among many other digestive-related etiologies. This is a side effect of faulty pancreatic enzyme secretion when lipid uptake is ineffective. The specialist performing the CF and parental carrier screening should be informed as a result of this discovery (9).

According to current recommendations, CF carrier screening should be provided to all women in the US who are pregnant or considering becoming pregnant, especially those of Caucasian ethnicity and/or those who have relatives with CF (10), taking into account the potential for distress if an affected child is born after carrier screening for negatives. As many infants as possible may be found to have a mutation by NBS (parental screening coming up negative), discrepancies may also arise between carrier screening panels and those used in NBS (11).

When a carrier is found, partner testing alternatives such as a variant panel or CFTR sequencing must be taken into account. Early results come from testing both partners of a relationship simultaneously with a panel (11, 12).

For carrier screening, hundreds of genes can be read simultaneously using the next-generation sequencing (NGS) technique. CF carrier screening, often referred to as expanded carrier screening, presents difficulties in this situation since it cannot disclose variations with unknown significance, and it still arises discussion regarding residual risk (12).

After using *in vitro* fertilization to create the embryos, carrier couples have the option of preimplantation genetic diagnosis (PGD/PIGD), which comprises embryo biopsy and genetic testing for known parental variations (12, 13). This gives the parents the option to select only those embryos for transfer that don't have the disease-causing gene-type. Choosing the outcome of a pregnancy coming from a damaged embryo is a true ethical turning point. As a result, carrier couples could have to make challenging decisions about whether to pursue PGD or, in the case of a current pregnancy, invasive prenatal diagnostic testing and (perhaps) abortion. When a fetus possesses a non-CF-causing mutation or a variant linked with a mild condition, appropriate counseling may be a turning point in parental decision regarding pregnancy ending (7, 13).

## 3 Newborn screening

Newborn CF screening is now a successful public health technique for early identification of afflicted infants, particularly in populations with high risk of CF prevalence (14). The majority of screening initiatives assess immuno-reactive trypsinogen (IRT) in dried blood spots, which must be taken within the first week of life. The next step is a second round of testing, which frequently entails molecular testing for CFTR mutations (13, 14). It has been obvious in recent years that adding DNA testing enhances specificity and speed, especially when two variations are found. Typically, the original sample is used for DNA testing, with several panels aimed at the population under examination. Given that newborns who carry the CF gene have higher IRT levels, this mindset may increase the likelihood of early recognition of CF carriers (14). A normal SCT (30 mmol/L) is utilized in the majority of screening programs to indicate a low likelihood of CF diagnosis. In some other protocols, the requirement for SCT is avoided by using a second IRT assessment at day-of-life 10–21 (assuming the result is normal) (15). Additionally, all European nations must actively implement neonatal CF screening because the newly developed molecularly targeted therapy regimens are most effective when used to manage pediatric patients.

## 4 CFTR genetic testing

Given that pediatric patients’ current treatment options are constantly being improved, a variety of diagnostic procedures are widely accessible. Even in the absence of a high sweat chloride test result, the presence of two causal variants is sufficient to diagnose CF; nevertheless, a minimum of two positive sweat chloride tests (SCT) is still required (16). Specialists have a variety of choices for genetic testing, as discussed below (17), depending on the family history, ethnicity, clinical characteristics, and the diagnosis given by the results of the newborn screening.

CFTR variant panel—identifies certain variations on the panel, frequently the most prevalent variants in the Caucasian population, but as a drawback, the method's detection rate varies greatly depending on the panel’s ethnic composition. It is primarily intended for use in newborn screening algorithms in developing nations (16, 17). It is also frequently employed in regular carrier screening in patients without a family history of CF (17).

Traditional sequencing (Sanger sequencing) can identify all sequence variations in CFTR's exons and intron-exon junctions, but it cannot identify significant duplications or deletions. It can also be performed as a carrier screen when one partner is affected or a known carrier (17). It is best appropriate for people with a CF diagnosis who are not Ashkenazi Jewish or European Caucasian, or who have fewer than two mutations discovered by panel test.

Deletion/duplication analysis. This method finds substantial deletions and duplications in CFTR that involve all or some of the exons, but it does not find sequence modifications. Individuals diagnosed with CF having less than two CF-causing mutations discovered after CFTR sequencing can benefit the most from this test (17).

Sequencing using the next generation. This test can identify sequence variations in CFTR's exons and intron-exon junctions, but it is unable to identify significant deletions or duplications without additional testing by specialized laboratories. This method can help people with CF diagnoses who are not in the ethnic risk group or who have fewer than two CF-causing mutations identified by panel testing. When one partner is afflicted or a known carrier, it may also be used as a carrier screen (17).

Targeted familial variant testing can be helpful for parent or sibling follow-up testing because it only detects one or two specific variants that have already been signalized in a family, as well as the presence or absence of certain variants in a patient's or carrier's family (17).

## 5 Pediatric screening

Genetic testing is now seen as a crucial step in collecting comprehensive diagnostic data for a baby or child with suspected CF. Since the molecular process of managing the etiology has been extensively researched (17, 18), the positive diagnosis can direct treatment at ever younger ages. Families are also impacted by this in terms of how they manage diagnostic, follow-up testing, and genetic counseling. According to the Cystic Fibrosis Foundation of the United States (CFF) standards, “families of infants diagnosed with CF should receive an appropriate education at the first diagnostic visit, and genetic counseling should be provided (18).”

First-degree siblings and half-siblings with particular CF symptoms should undergo an SCT to start the investigation into the patient's family members (18, 19). Most clinicians suggest an SCT, but some also support family variant testing in place of or in addition to an SCT (19). It is necessary to rule out the possibility that the sibling is a carrier if the SCT value is negative because there is still a 2/3 (66%) chance that they are. Genetic counseling is necessary both before and after carrier testing if it is to be done. Special attention should be paid to the diagnostic capabilities and constraints of both SCT and family variant tests when the proband's CF genotype is linked to variable SCT levels 60 mmol/L (as in the case of the R117H specific mutation) (19).

## 6 Treatment and management of children with CF

In order to maintain growth and nutrition in CF patients, it is necessary to administer mucus thinner, clean the airways, and antibiotic support when requested by the biological and clinical context (20).

It is important to review and refine CF treatment plans while closely monitoring patients to ensure early and proactive comorbidity management. Prospective CF patients should be monitored in order to meet these objectives, even before the diagnosis is made and more information is gathered (21). Once the child has been diagnosed, the clinicians should immediately begin the patient's treatment and inform the patient's family on how to handle the illness. The most recent advancements in CF care include molecularly generated medications that are highly effective and provide an individualized strategy for patients of all ages. These medications are based on the disease's pathogenetic pathway (22).

### 6.1 CF treatment regimens

Inhalation therapy involves hydrating mucus in CF patient airways with hypertonic saline solution vapors, in order to achieve a thin and efficient layer of mucus. This therapeutic approach is employed from birth and has the advantage of having few side effects. In order to recreate the water-containing surface layer that is missing in CF patients, the high osmotic pressure of the solution can drag water out of the airway epithelial cells (23). Numerous products are already available on the market with specific instructions for daily usage. Only having the mechanical effect of cleaning the airway of thick mucus, these solutions should be enhanced with other active substances. Numerous studies have examined the therapeutic value of bronchodilator medication in CF patients, but none have found a significant improvement in the airway clearance (24).

Chest physiotherapy is the recommended treatment for clearing secretions in patients who have continued retention of mucus and purulent secretions that restrict airflow and harm airways. The most efficient mechanical approach utilized for this is physiotherapy, which relies on postural drainage and percussion, sometimes in conjunction with bronchoscopy lavage (25).

Antibiotics are necessary for the treatment of chronic infections and acute CF exacerbations in certain situations with a high risk of infection and in individuals with bacterial colonization. In general, long-term oral antibiotic regimens are not advised for infection control since children could acquire medication resistance. This is the rationale behind the long-term use of aerosolized antibiotics, typically tobramycin and aztreonam, which are advised due to their favorable effects on lung function and are typically used to treat Pseudomonas aeruginosa and the removal of germs. However, due to its anti-inflammatory and antibacterial qualities, long-term azithromycin treatment is still advised for young CF patients (26).

The formation of biofilms permits the proliferation and adaptation of bacteria in anoxic and nutrient-poor settings, in addition to offering protection from the host immune system and/or antimicrobial medications (27). Biofilms contain an unexpectedly large number of bacterial sub-communities, each with a different degree of metabolic activity. In contrast, low or nonexistent metabolic activity in inner subpopulations makes them more resistant to antimicrobial drugs, which causes infection persistence and/or recurrence (28). High metabolic activity in peripheral subpopulations causes them to consume a lot of oxygen and nutrients. Higher dosages of antibiotics or liposomal antibiotic formulations may be more effective, despite the increased risk of toxicity and AMR (29).

The cornerstone of CF management is usually antibiotics; patients get repeated doses of broad-spectrum with the intention of increasing their lifespan and raising their standard of living. Antibiotics are utilized in the management of recurring or chronic infections, the treatment of pulmonary exacerbations, and the early elimination of Pseudomonas aeruginosa (30). Depending on the organism's *in vitro* susceptibility, it's critical to begin an appropriate antibiotic therapy as soon as feasible in order to treat Pseudomonas aeruginosa infections. By doing this, major problems and morbidity may be avoided (31). Nonetheless, aggressive antibiotic therapy lowers the bacterial burden but often makes the eradication of a persistent lung infection unsuccessful (32).

The presence of AMR is a major health concern for those with CF. In this regard, controlling and keeping an eye on the use of antibiotics is crucial, now more than ever. When it comes to treating strains of Pseudomonas aeruginosa, Staphylococcus aureus (especially MRSA), Burkholderia cepacia complex, and Achromobacter that are frequently isolated in the respiratory tract of these patients and typically develop AMR, the right course of action is to target the underlying resistance mechanisms. Consequently, there has been an exponential rise in interest in emerging molecular-based diagnostic techniques. Furthermore, because the development of new medicines is a delayed process, it is critical to create effective treatment strategies to eradicate recurrent infections in people with cystic fibrosis, requiring a multidisciplinary effort (33).

Patients with CF, especially children, need to have their specific growth and nutritional plans regularly evaluated and supported. Pancreatic enzymes that are estimated based on daily lipid intake, along with a high intake of calories, and support for fat-soluble vitamins, should be included in the daily diet of CF patients (34). To construct a daily diet plan, these particular categories of patients require the involvement of a nutritionist, keeping in mind the increased risk of acquiring diabetes mellitus. This is necessary for kids, whose growth shouldn't be limited or hampered by food choices (34).

A number of novel medication classes are being developed, some of which are well tolerated by pediatric patients. These drug classes include those that work directly to affect mucociliary clearance as well as those that fix damaged CFTR protein function (34). The CFTR modulators, which comprise correctors, potentiators, stabilizers, amplifiers, and read-through agents, target the protein's synthesis, processing, or expression (35). This therapy strategy is called “targeted” or “mutation specific” since the kind of molecules that patients receive depend on the CFTR mutations they have (36).

With notable improvements in biological and clinical endpoints of CF (such as sweat chloride concentration or FEV1), numerous authors have explored the possible benefits of modulator treatments during the past ten years (36, 37). Deciphering a new, personalized, and enhanced therapeutic approach was made possible by understanding the molecular foundation of the pathogenetic pathway in CF. Initially, only adult patients could benefit from those treatment plans, but at this point, medical professionals are also using novel therapeutic approaches with pediatric patients, completing the hunt for pharmaceutical cures meant to solve the CFTR protein abnormality (37).

Ivacaftor—Kalydeco® (a molecular potentiator), the first CFTR modulator, was authorized in 2012 for the treatment of CF patients aged 6 years with at least one G551D mutation. This was over 25 years after the CFTR gene was discovered. Ivacaftor is a small-molecule CFTR function potentiator that improves chloride transport in both wild-type and several mutant CFTR forms *in vitro*, including the G551D mutation (38) by boosting protein channel gating. First, adults with CF have been shown to benefit clinically from ivacaftor, which has also been shown to enhance nutritional status and lung function in recent studies in children over the age of six (31–33). As studies continue to demonstrate Ivacaftor's safety in newborns, it had also improved biomarkers of exocrine pancreatic function in children aged 1–5 (39–41). For instance, the ARRIVAL study evaluated the safety and tolerability of ivacaftor in CF groups aged 12–24 months. Ivacaftor can be safely dosed in infants under 4 months of age, according to the author's findings, which are consistent with observations in older children (42). Ivacaftor's safety profile matched the safety profile that had previously been developed. Significant increases in sweat chloride levels are indicative of better CFTR performance. Additionally, improvements in pancreatic function markers point to ivacaftor's potential to prevent or slow the progression of exocrine pancreatic dysfunction. As a result, the specialists established that the underlying molecular etiology of CF in infants less than 4 months can be effectively and safely treated with ivacaftor (42).

Current findings in young children suggest that early intervention with CFTR modulators may delay or slow the progression of exocrine pancreatic insufficiency and impaired growth, which are, basically, the main goal in managing this category of patients (43).

The combination of a potentiator-ivacaftor and a corrector-lumacaftor (lumacaftor/ivacaftor—Orkambi®) was approved for pediatric patients aged 12 years who were homozygous for the p.Phe508del gene in 2015 (43, 44). This combination was also linked to a lower rate of pulmonary exacerbations, hospitalizations, and usage of intravenous antibiotics, dramatically increasing the percentage of predicted FEV1 (from 2.6 to 4.0 points). Tezacaftor/ivacaftor—Symdeko®—a second dual combination first introduced in 2018 (45, 46).

**Figure 1:**
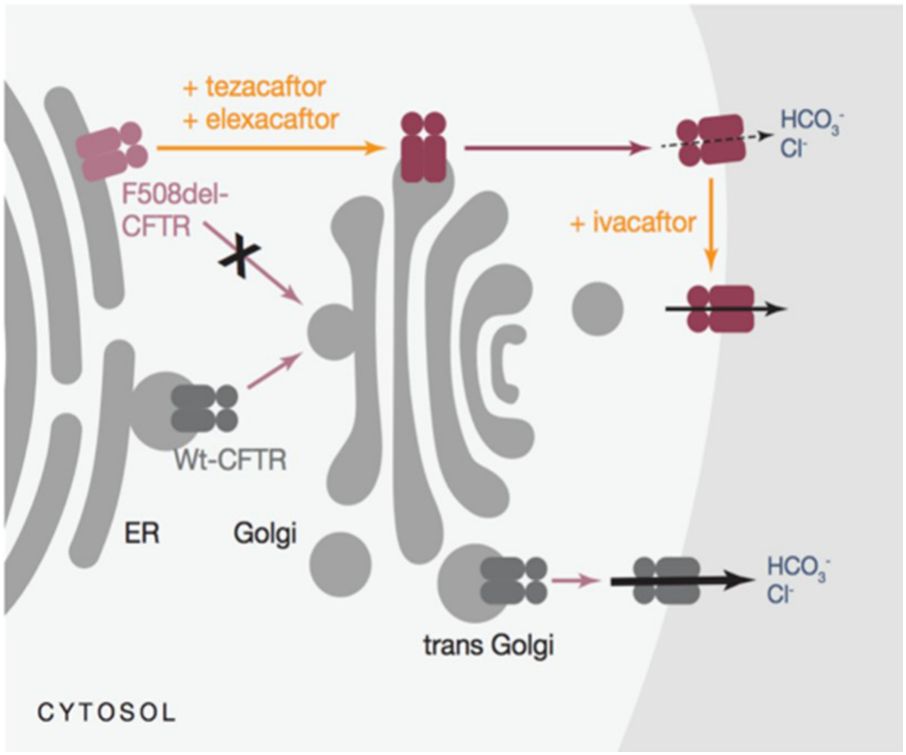
Mechanism of action for triKafta (47).

A triple combination therapy (elexacaftor/tezacaftor/ivacaftor-TrikaftaTM) was recently licensed initially for the treatment of patients aged 12 years bearing at least one p.Phe508del mutation (47) (Figure 1). This extremely promising associative therapy led to considerable improvements in sweat chloride concentration, pulmonary exacerbations, and patient and family quality of life in addition to an increase of up to 14 points in the percentage of predicted FEV1. In individuals with CF and one or more F508del alleles, it exhibits safety and sustained efficacy for 24 weeks or longer (48, 49).

It is currently too early to determine these compounds’ effects on patient survival because there is variation in response and they have just been on the market since 2012. It is obvious that targeted medicines are improving the lives of CF patients, and in the years to come, it is anticipated that their introduction will significantly increase patient survival. Early treatment beginning and treatment persistence in the absence of patient and family cooperation considerably increase the chance of survival (48, 49).

To prevent long-term infection and inflammation that eventually cause irreversible bronchiectasis and respiratory failure, lung transplantation is feasible for end-stage patient treatment depending on the health of the particular patient (50).

### 6.2 Developing novel therapies

As a result, over the past ten years, the focus of CF treatment has changed from symptomatic management to CFTR protein function restoration. The discipline of personalized or precision medicine has grown thanks to these tailored medicines (51). New drugs will penalize other categories of medication, especially those with the rarest of variants, while allegedly benefiting patients with specific mutations. With DNA or mRNA replacement techniques, the current stage of “mutation- agnostic” therapies suitable for all CF patients could be reached (51).

Recently, oligonucleotides to repair CFTR mRNA were in focus to be studied regarding clinical response and safety in adult patients. Eluforsen, is an agent containing a 33 base antisense oligonucleotide (ASO) targeting the most frequent mutation, p.Phe508del CFTR. Electrophysiological assays conducted both *in vitro* and in animal studies showed restoration of CFTR function. In actual human trials, Eluforsen was well tolerated, with a promising safety profile (51).

The focus is on tiny compounds that can speed up premature truncation codon (PTC) read-through and/or prevent mRNA decay for patients with class I (nonsense) mutations. With this technique, the patient can have clinically significant amounts of functioning CFTR. Because of nonsense-mediated decay (NMD) mechanisms, PTCs cause short-lived mRNA (52). When creating a personalized treatment plan, ribosomal read-through agents were also taken into account. Ataluren was the most developed and studied, and early trials and preclinical study outcomes were encouraging (53).

Ultimately, Ataluren failed to demonstrate clinical benefit in larger phase three trials. An alternative strategy to overcome PTC mutations is engineered transfer RNAs (tRNAs). These drugs are designed to introduce an amino acid to an elongating peptide in place of the termination codon (53, 54).

Direct distribution of the medication to the respiratory epithelium is the main difficulty faced by novel medicines generated from genetics. Translate Bio (which is aerosolized for inhalation) is an ongoing clinical experiment examining the potential of mRNA delivery for CF patients. It makes use of a unique lipid-based nanoparticle carrier for mRNA delivery (MRT5005). The initial findings showed that patients’ ameliorating ppFEV1 had changed by at least 10%, with the potential to expand findings within a therapy cohort (54).

Pharmaceutical developers are currently collaborating on a first-in-man trial using a pseudo-typed lentiviral vector at the preclinical stage (55). In phase I and pre-clinical stages, respectively, 4D Molecular Therapeutics and Spirovant are employing adeno-associated vectors to transfer CFTR DNA. There have been various clinical trials using viral and non-viral methods for gene (DNA) transfer up to this point, the bulk of which were created as early phase proof of concept studies without clinical efficacy read-outs (55, 56).

### 6.3 Drugs targeting CF inflammation status

Despite having a considerable impact on lung function, it is unknown how CFTR modulators affect pulmonary inflammation. Extensive research has been done on the fascinating notion that lung tissue can heal itself when CFTR function has been restored. It is generally known that healthy lung tissue may regenerate after injury, and that this process depends on the activity of basal cells in the airway, which operate as resident lung stem cells and multiply and differentiate in response to injury. The reduction of inflammation is a crucial first step in the process of tissue repair, and it may be facilitated by the interaction of immune cells, such as malfunctioning macrophages, with local stem cells (57, 58).

There is unquestionably proof that inflammation continues even when the respiratory epithelium’s CFTR activity is restored (58). Numerous targets for the creation of new medications can be found in the presence of increased neutrophilia, pro-inflammatory macrophages, and a variety of pro-inflammatory mediators (59). Drugs that directly address CF inflammation remain elusive in people over the age of 50, despite decades of ongoing study. It is claimed that early and persistent neutrophil influx and high levels of elastase, which are associated with structural damage even in infancy, are features of the inflammatory process in CF. Studies have shown that typical processes for resolution are compromised (59), causing inflammation to continue even after an infective assault has been resolved.

Leukotriene B4 (LTB4), a neutrophil chemoattractant, was thought to be a promising therapeutic target that might be used to develop novel drugs by blocking neutrophil recruitment to the lung. Amebulant, an LTB4 antagonist, showed promise in preclinical testing but failed in human trials because it was linked to more pulmonary exacerbations in a phase II study (60). Acebilustat, an LTA4 hydroxylase inhibitor, can lower LTB4 levels as opposed to totally preventing LTB4's effects. The agent led to reduced lung neutrophil levels and exhibited a good safety profile in early phase studies (60). The cannabinoid receptor agonist, Lenabasum can reduce IL-6 transcription in macrophages *in vitro* (61), suggesting direct effects on the inflammatory potential of these cells. It had modest clinical effects, but exhibited a significant reduction in sputum interleukin-8 content (61).

Airway infection (bacterial, mycobacterial, and fungal) is a life-limiting condition that affects CF patients of all ages throughout their lifetimes (62). Because the arsenal of medications available to treat juvenile patients is constrained in terms of dosage, scope, and efficacy, more novel drugs must be created to treat these widespread species. Pathogens that are rarer but more difficult to treat, like the non-tuberculous mycobacterium (NTM). M. abscessus (61, 62), can cause acute and chronic problems that have a negative impact on the prognosis of infected individuals. A wide range of medications, including antibiotic adjuvants, biofilm-targeted strategies, and bacteriophages, are waiting to be approved for use in pediatric pathology related to CF (63).

### 6.4 Future therapeutic approaches

Since they may not already have irreversible organ dysfunction, infants and children with CF are thought to be the population group most likely to benefit from novel treatments for CF (63, 64). Their benefit in this aspect is also a drawback because it is challenging to evaluate improvement in any outcome measure from a typical baseline, especially in very young children. Children beyond the age of three can perform LCI and several imaging tests, providing a more accurate assessment of the therapy benefit (63). Although efficacy was extrapolated from older cohorts, FDA approval for ivacaftor in the youngest cohorts was based on pharmacokinetics, pharmacodynamics (sweat chloride), and safety. Future studies of systemic CFTR-targeted therapies should incorporate this assay as long as significant alterations in fecal elastase, a biomarker of pancreatic exocrine function, have been shown in pediatric patients with a high likelihood of receiving a positive CF diagnosis (64, 65).

Giving the importance of an effective clearance of the lungs, airway surface rehydration and reducing mucus viscosity are additional targets of new therapeutic approaches. Sodium channel (ENaC) blockers have been under development and study for the last years, but until now no agent has yet progressed through pivotal trials to licensing (63–65). An agonist of an alternative chloride channel, TMEM16A, (EDT002) is in early phase trials. Clinical trials are currently being conducted on oligonucleotides such OligoG, a seaweed-derived substance that acts on both bacterial biofilms and mucus (66). It is unclear whether patients who are receiving significant health benefits from CFTR modulators will still require these medications, but there is undoubtedly a need in those cases. Additionally, these medications may be helpful for conditions other than CF such as other types of bronchiectasis and chronic obstructive pulmonary disease (COPD), for which there are currently no effective treatments (64–67).

Corrector therapy is a priority, and additional approaches to slowing the spread of the illness are being investigated (68). As a potential corrective method for people with F508del-CFTR, the direct and indirect regulation of the nitric oxide (NO) pathway has recently received attention (69). The most recent medication, Riociguat, is an oral NO-independent soluble guanylate cyclase (sGC) stimulator. Riociguat is now licensed for use in patients with idiopathic pulmonary hypertension and chronic thromboembolic pulmonary hypertension (CTEPH). It does this by increasing the sensitivity of sGC to NO and increasing the synthesis of cyclic guanosine monophosphate. This GC stimulator's safety, tolerability, and efficacy objectives in CF patients are still being evaluated (70).

**Figure 2:**
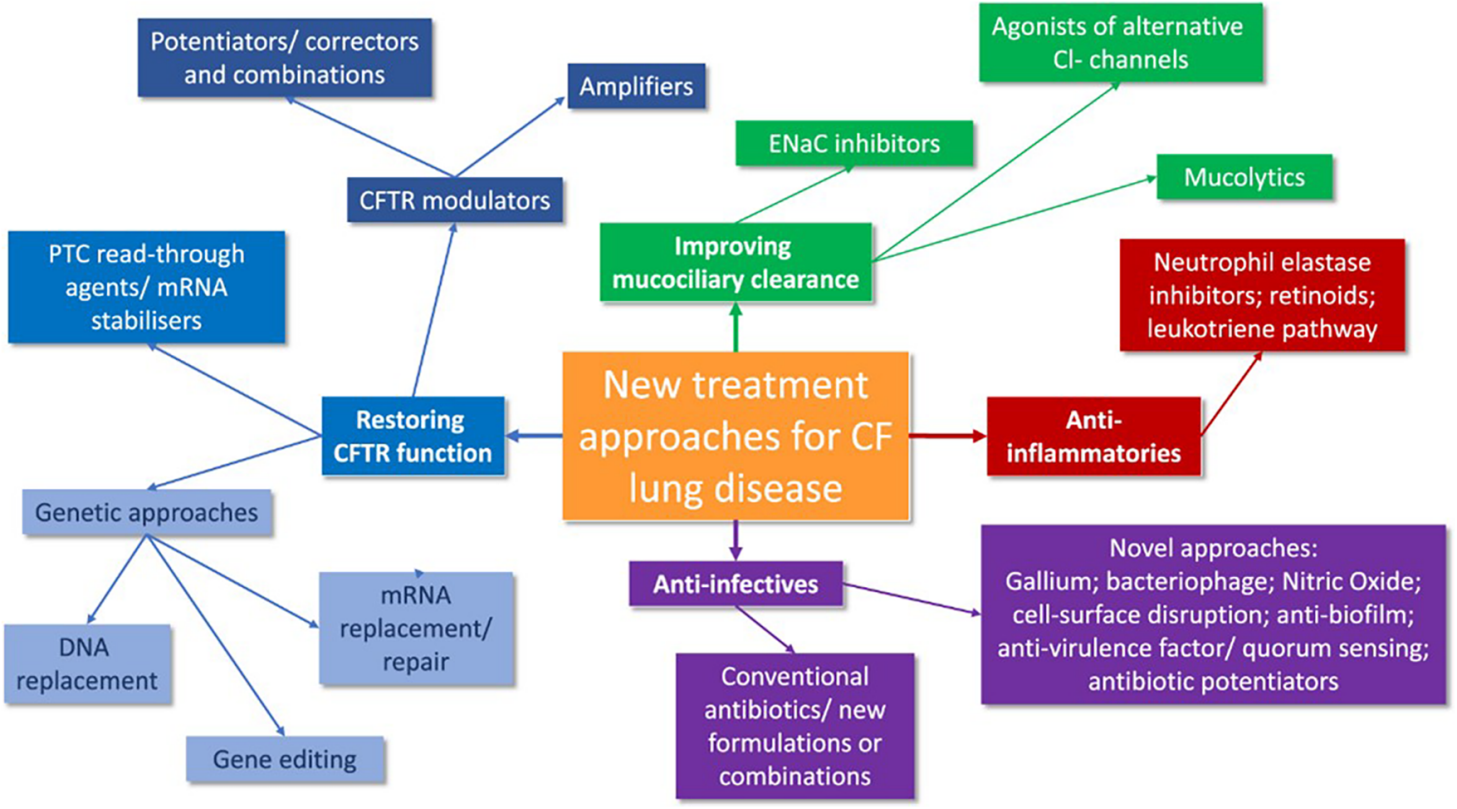
A synthesis of future CF treatment approaches (77).

Similarly, a novel class of molecules that indirectly increases epithelial and smooth muscle NO via inhibition of S-nitrosoglutathione reductase (GSNOR), the enzyme which degrades S-nitrosoglutathione (GSNO), are emerging for approval (71). GSNOR inhibitor compounds have been shown to increase cell surface localized Phe508del-CFTR and CFTR activity in human bronchial epithelial cells and in a murine model of CF *in vivo*, possibly via reduction of chaperone-protein direction towards ERAD-mediated degradation of Phe508del CFTR protein (71–73). Safety and tolerability in patients aged 18 years and older were demonstrated recently, since a Phase 2a, placebo- controlled study of adults CF patients homozygous for F508del-CFTR has been currently extended for results (73–76). The canvas of new treatment approaches in CF is synthetically expressed in Figure 2 (77).

Many individuals cannot benefit from current modulator therapy because their specific CFTR mutations do not lend themselves to the “making the most of a mutant protein” strategy that is the cornerstone of modern modulator therapy. For these patients, other therapies such ribosomal readthrough agents and RNA-specific tactics [transfer (t)RNAs, mRNA stabilizers, and repair] are needed. While mRNA and DNA replacement are currently undergoing clinical trials, gene editing remains an active area of research (78). However, there are problems in testing children for genetic therapy, which adds to the delay in specific therapy in pediatric practice.

It may be possible to create “mutation agnostic” treatments using DNA or mRNA replacement methods for every CF patient. Its primary barrier is delivery to the respiratory epithelium. There have been two interim results in the past 18 months from a clinical trial investigating the possibility of mRNA delivery for cystic fibrosis. Translate Bio uses an aerosolized lipid-based nanoparticle carrier (MRT5005) that is meant to be inhaled to deliver mRNA. Many clinical trials using viral and non-viral gene transfer techniques have been conducted thus far; however, the vast majority of these investigations were designed as early phase proof of concept studies without clinical efficacy read-outs (78). 4D Molecular Therapeutics and Spirovant use adeno-associated vectors to transfer CFTR DNA, and they are currently in phase I and pre-clinical stages, respectively, but only in adult patients, without being standardized in pediatric practice.

## 7 Conclusions

Since there have been more than 1,000 rare mutations found worldwide, treating the rare disease CF has become extremely difficult. Therefore, the diagnosis of CF in such situations necessitates the adoption of novel approaches as conventional clinical procedures are not appropriate for detecting these rare mutations. However, in order to better direct patient therapy and enhance patient outcomes, it is critical to study uncommon CF mutations, which can offer crucial information about illness prognosis and genotype-phenotype connections. To ensure the success of developing newer, safer, and more successful treatment options, a deeper understanding of the pathogeny of the disease is required. Such studies will be crucial for enhancing patient care and quality of life in this era of individualized medicine and treatment.

## References

[1] Bell SC Mall MA Gutierrez H Macek M Madge S Davies JC The future of cystic fibrosis care: a global perspective. Lancet Respir Med. (2020) 8(1):65–124. 10.1016/S2213-2600(19)30337-6 31570318 PMC8862661

[2] McBennett KA Davis PB Konstan MW. Increasing life expectancy in cystic fibrosis: advances and challenges. Pediatr Pulmonol. (2022) 57(1):S5–S12. 10.1002/ppul.25733 34672432 PMC9004282

[3] Bierlaagh MC Muilwijk D Beekman JM van der Ent CK. A new era for people with cystic fibrosis. Eur J Pediatr. (2021) 180(9):2731–9. 10.1007/s00431-021-04168-y 34213646 PMC8346380

[4] Dana J Debray D Beaufrère A Hillaire S Fabre M Reinhold C Cystic fibrosis-related liver disease: clinical presentations, diagnostic and monitoring approaches in the era of CFTR modulator therapies. J Hepatol. (2022) 76(2):420–34. 10.1016/j.jhep.2021.09.042 34678405

[5] Vastag B. Cystic fibrosis gene testing a challenge. JAMA. (2003) 289(22):2923–4. 10.1001/jama.289.22.2923 12799389

[6] Quintana-Gallego E Delgado-Pecellín I Calero Acuña C. CFTR protein repair therapy in cystic fibrosis. Arch Bronconeumol. (2014) 50(4):146–50. 10.1016/j.arbres.2013.07.013 24095197

[7] Foil KE Powers A Raraigh KS Wallis K Southern KW Salinas D. The increasing challenge of genetic counseling for cystic fibrosis. J Cyst Fibros. (2019) 18(2):167–74. 10.1016/j.jcf.2018.11.014 30527892

[8] Sukupová M Dhaifalah I Adamík Z Havalová J. Hyperechogenita intestina jako marker cystické fibrózy u plodu (hyperechogenic fetal bowel as a marker of fetal cystic fibrosis). Ceska Gynekol. (2015) 80(1):20–4. 25723074

[9] Muller F Simon-Bouy B Girodon E Monnier N Malinge MC Serre JL. Predicting the risk of cystic fibrosis with abnormal ultrasound signs of fetal bowel: results of a French molecular collaborative study based on 641 prospective cases. Am J Med Genet. (2002) 110(2):109–15. 10.1002/ajmg.10431 12116247

[10] Barben J Castellani C Munck A Davies JC de Winter-de Groot KM Gartner S Southern KW; European CF society neonatal screening working group (ECFS NSWG). updated guidance on the management of children with cystic fibrosis transmembrane conductance regulator-related metabolic syndrome/cystic fibrosis screen positive, inconclusive diagnosis (CRMS/CFSPID). J Cyst Fibros. (2021) 20(5):810–9. 10.1016/j.jcf.2020.11.006 33257262

[11] Castellani C Picci L Tridello G Casati E Tamanini A Bartoloni L Cystic fibrosis carrier screening effects on birth prevalence and newborn screening. Genet Med. (2016) 18(2):145–51. 10.1038/gim.2015.68 26087173

[12] D’Alcamo E Gullo G Cucinella G Perino A Burgio S Etrusco A Cystic fibrosis assessment in infertile couples: genetic analysis trough the next generation sequencing technique. Clin Exp Obstet Gynecol. (2022) 49(5):105. 10.31083/j.ceog4905105

[13] Ciske DJ Haavisto A Laxova A Rock LZ Farrell PM. Genetic counseling and neonatal screening for cystic fibrosis: an assessment of the communication process. Pediatrics. (2001) 107(4):699–705. 10.1542/peds.107.4.699 11335747

[14] Davies G. Does newborn screening improve early lung function in cystic fibrosis? Paediatr Respir Rev. (2022) 42:17–22. 10.1016/j.prrv.2020.08.005 32952050

[15] Baykal NK Aslan AT Asfuroglu P Ersoy A Kose M Unal G P180 evaluation of clinical features of children with cystic fibrosis and CFSPID in newborn screening programme with IRT/IRT protocol. J Cyst Fibros. (2023) 22:S119–20. 10.1016/S1569-1993(23)00555-6

[16] Kessels SJM Carter D Ellery B Newton S Merlin TL. Prenatal genetic testing for cystic fibrosis: a systematic review of clinical effectiveness and an ethics review. Genet Med. (2020) 22(2):258–67. 10.1038/s41436-019-0641-8 31467445

[17] Pagin A Sermet-Gaudelus I Burgel PR. Genetic diagnosis in practice: from cystic fibrosis to CFTR-related disorders. Arch Pediatr. (2020) 27(1):eS25–9. 10.1016/S0929-693X(20)30047-6 32172933

[18] Cutting GR. Cystic fibrosis genetics: from molecular understanding to clinical application. Nat Rev Genet. (2015) 16(1):45–56. 10.1038/nrg3849 25404111 PMC4364438

[19] Corvol H Flamant C Vallet C Clement A Brouard J. Les gènes modificateurs dans la mucoviscidose (modifier genes and cystic fibrosis). Arch Pediatr. (2006) 13(1):57–63. 10.1016/j.arcped.2005.09.029 16274977

[20] Sunman B Ademhan Tural D Ozsezen B Emiralioglu N Yalcin E Özçelik U. Current approach in the diagnosis and management of allergic bronchopulmonary aspergillosis in children with cystic fibrosis. Front Pediatr. (2020) 8:582964. 10.3389/fped.2020.582964 33194914 PMC7606581

[21] Le TN Anabtawi A Putman MS Tangpricha V Stalvey MS. Growth failure and treatment in cystic fibrosis. J Cyst Fibros. (2019) 18(2):S82–7. 10.1016/j.jcf.2019.08.010 31679733 PMC6934044

[22] Somayaji R Nichols DP Bell SC. Cystic fibrosis—ten promising therapeutic approaches in the current era of care. Expert Opin Investig Drugs. (2020) 29(10):1107–24. 10.1080/13543784.2020.1805733 32744089

[23] Dellon EP Donaldson SH Johnson R Davis SD. Safety and tolerability of inhaled hypertonic saline in young children with cystic fibrosis. Pediatr Pulmonol. (2008) 43(11):1100–6. 10.1002/ppul.20909 18828160

[24] Reeves EP Molloy K Pohl K McElvaney NG. Hypertonic saline in treatment of pulmonary disease in cystic fibrosis. ScientificWorldJournal. (2012) 2012:465230. 10.1100/2012/465230 22645424 PMC3356721

[25] Elbasan B Tunali N Duzgun I Ozcelik U. Effects of chest physiotherapy and aerobic exercise training on physical fitness in young children with cystic fibrosis. Ital J Pediatr. (2012) 38:2. 10.1186/1824-7288-38-2 22233967 PMC3269357

[26] Ciuca IM Dediu M Popin D Pop LL Tamas LA Pilut CN Antibiotherapy in children with cystic fibrosis-an extensive review. Children (Basel). (2022) 9(8):1258. 10.3390/children9081258 36010149 PMC9406924

[27] Bugli F Martini C Di Vito M Cacaci M Catalucci D Gori A Antimicrobial peptides for tackling cystic fibrosis related bacterial infections: a review. Microbiol. Res. (2022) 263:127152. 10.1016/j.micres.2022.127152 35944357

[28] Stewart PS Zhang T Xu R Pitts B Walters MC Roe F Reaction-diffusion theory explains hypoxia and heterogeneous growth within microbial biofilms associated with chronic infections. NPJ Biofilms Microbiomes. (2016) 2:16012. 10.1038/npjbiofilms.2016.12 28721248 PMC5515263

[29] Hengzhuang W Wu H Ciofu O Song Z Høiby N. In vivo pharmacokinetics/pharmacodynamics of colistin and imipenem in Pseudomonas aeruginosa biofilm infection. Antimicrob Agents Chemother. (2012) 56:2683–90. 10.1128/AAC.06486-11 22354300 PMC3346607

[30] Van den Bossche S De Broe E Coenye T Van Braeckel E Crabbé A. The cystic fibrosis lung microenvironment alters antibiotic activity: causes and effects. Eur Respir Rev. (2021) 30:210055. 10.1183/16000617.0055-2021 34526313 PMC9489179

[31] Kunz Coyne AJ Alshaer M Casapao AM Venugopalan V Isache C Ferreira J Effectiveness and safety of Beta-lactam antibiotics with and without therapeutic drug monitoring in patients with Pseudomonas aeruginosa pneumonia or bloodstream infection. Antimicrob Agents Chemother. (2022) 66:e0064622. 10.1128/aac.00646-22 36073943 PMC9578394

[32] Bonyadi P Saleh NT Dehghani M Yamini M Amini K. Prevalence of antibiotic resistance of Pseudomonas aeruginosa in cystic fibrosis infection: a systematic review and meta-analysis. Microb Pathog. (2022) 165:105461. 10.1016/j.micpath.2022.105461 35240288

[33] Perikleous EP Gkentzi D Bertzouanis A Paraskakis E Sovtic A Fouzas S. Antibiotic resistance in patients with cystic fibrosis: past. present, and future. Antibiotics. (2023) 12:217. 10.3390/antibiotics12020217 36830128 PMC9951886

[34] Haupt ME Kwasny MJ Schechter MS McColley SA. Pancreatic enzyme replacement therapy dosing and nutritional outcomes in children with cystic fibrosis. J Pediatr. (2014) 164(5):1110–1115.e1. 10.1016/j.jpeds.2014.01.022 24560182

[35] Scotet V L'Hostis C Férec C. The changing epidemiology of cystic fibrosis: incidence. Survival and Impact of the CFTR Gene Discovery. Genes (Basel). (2020) 11(6):589. 10.3390/genes11060589 32466381 PMC7348877

[36] Balfour-Lynn IM King JA. CFTR modulator therapies—effect on life expectancy in people with cystic fibrosis. Paediatr Respir Rev. (2022) 42:3–8. 10.1016/j.prrv.2020.05.002 32565113 PMC7255286

[37] Nichols DP Donaldson SH Frederick CA Freedman SD Gelfond D Hoffman LR PROMISE: working with the CF community to understand emerging clinical and research needs for those treated with highly effective CFTR modulator therapy. J Cyst Fibros. (2021) 20(2):205–12. 10.1016/j.jcf.2021.02.003 33619012 PMC8686210

[38] Van Goor F Yu H Burton B Hoffman BJ. Effect of ivacaftor on CFTR forms with missense mutations associated with defects in protein processing or function. J Cyst Fibros. (2014) 13(1):29–36. 10.1016/j.jcf.2013.06.008 23891399

[39] Flume PA Liou TG Borowitz DS Li H Yen K Ordoñez CL Ivacaftor in subjects with cystic fibrosis who are homozygous for the F508del-CFTR mutation. Chest. (2012) 142(3):718–24. 10.1378/chest.11-2672 22383668 PMC3435140

[40] Yu H Burton B Huang CJ Worley J Cao D Johnson JP Jr Ivacaftor potentiation of multiple CFTR channels with gating mutations. J Cyst Fibros. (2012) 11(3):237–45. 10.1016/j.jcf.2011.12.005 22293084

[41] Sermet-Gaudelus I. Ivacaftor treatment in patients with cystic fibrosis and the G551D-CFTR mutation. Eur Respir Rev. (2013) 22(127):66–71. 10.1183/09059180.00008512 23457167 PMC9487423

[42] Rosenfeld M Wainwright CE Higgins M Wang LT McKee C Campbell D Ivacaftor treatment of cystic fibrosis in children aged 12 to <24 months and with a CFTR gating mutation (ARRIVAL): a phase 3 single-arm study. Lancet Respir Med. (2018) 6(7):545–53. 10.1016/S2213-2600(18)30202-9 29886024 PMC6626762

[43] Lopes-Pacheco M. CFTR modulators: the changing face of cystic fibrosis in the era of precision medicine. Front Pharmacol. (2020) 10:1662. 10.3389/fphar.2019.01662 32153386 PMC7046560

[44] Shteinberg M Taylor-Cousar JL. Impact of CFTR modulator use on outcomes in people with severe cystic fibrosis lung disease. Eur Respir Rev. (2020) 29(155):190112. 10.1183/16000617.0112-2019 32198216 PMC9488599

[45] Laselva O Bartlett C Popa A Ouyang H Gunawardena TNA Gonska T Emerging preclinical modulators developed for F508del-CFTR have the potential to be effective for ORKAMBI resistant processing mutants. J Cyst Fibros. (2021) 20(1):106–19. 10.1016/j.jcf.2020.07.015 32741662

[46] Cuevas-Ocaña S Laselva O Avolio J Nenna R. The era of CFTR modulators: improvements made and remaining challenges. Breathe (Sheff). (2020) 16(2):200016. 10.1183/20734735.0016-2020 33304402 PMC7714553

[47] Zaher A ElSaygh J Elsori D ElSaygh H Sanni A. A review of trikafta: triple cystic fibrosis transmembrane conductance regulator (CFTR) modulator therapy. Cureus. (2021) 13(7):e16144. 10.7759/cureus.16144 34268058 PMC8266292

[48] Bear CE. A therapy for most with cystic fibrosis. Cell. (2020) 180(2):211. 10.1016/j.cell.2019.12.032 31978337

[49] Jordan KD Zemanick ET Taylor-Cousar JL Hoppe JE. Managing cystic fibrosis in children aged 6–11yrs: a critical review of elexacaftor/tezacaftor/ivacaftor combination therapy. Expert Rev Respir Med. (2023) 17(2):97–108. 10.1080/17476348.2023.2179989 36803356

[50] Davies JC Wainwright CE Sawicki GS Higgins MN Campbell D Harris C Ivacaftor in infants aged 4 to <12 months with cystic fibrosis and a gating mutation. Results of a two-part phase 3 clinical trial. Am J Respir Crit Care Med. (2021) 203(5):585–93. 10.1164/rccm.202008-3177OC 33023304 PMC7924576

[51] Sermet-Gaudelus I Clancy JP Nichols DP Nick JA De Boeck K Solomon GM Antisense oligonucleotide eluforsen improves CFTR function in F508del cystic fibrosis. J Cyst Fibros. (2019) 18(4):536–42. 10.1016/j.jcf.2018.10.015 30467074 PMC7227803

[52] Brinks V Lipinska K de Jager M Beumer W Button B Livraghi-Butrico A The cystic fibrosis-like airway surface layer is not a significant barrier for delivery of eluforsen to airway epithelial cells. J Aerosol Med Pulm Drug Deliv. (2019) 32(5):303–16. 10.1089/jamp.2018.1502 31120356 PMC6781260

[53] Pibiri I Lentini L Melfi R Gallucci G Pace A Spinello A Enhancement of premature stop codon readthrough in the CFTR gene by ataluren (PTC124) derivatives. Eur J Med Chem. (2015) 101:236–44. 10.1016/j.ejmech.2015.06.038 26142488

[54] Wilschanski M Miller LL Shoseyov D Blau H Rivlin J Aviram M Chronic ataluren (PTC124) treatment of nonsense mutation cystic fibrosis. Eur Respir J. (2011) 38(1):59–69. 10.1183/09031936.00120910 21233271

[55] Krishnamurthy S Traore S Cooney AL Brommel CM Kulhankova K Sinn PL Functional correction of CFTR mutations in human airway epithelial cells using adenine base editors. Nucleic Acids Res. (2021) 49(18):10558–72. 10.1093/nar/gkab7882 34520545 PMC8501978

[56] Christopher Boyd A Guo S Huang L Kerem B Oren YS Walker AJ New approaches to genetic therapies for cystic fibrosis. J Cyst Fibros. (2020) 19(1):S54–9. 10.1016/j.jcf.2019.12.012 31948871

[57] Elizur A Cannon CL Ferkol TW. Airway inflammation in cystic fibrosis. Chest. (2008) 133(2):489–95. 10.1378/chest.07-1631 18252915

[58] Courtney JM Ennis M Elborn JS. Cytokines and inflammatory mediators in cystic fibrosis. J Cyst Fibros. (2004) 3(4):223–31. 10.1016/j.jcf.2004.06.006 15698939

[59] Cohen-Cymberknoh M Kerem E Ferkol T Elizur A. Airway inflammation in cystic fibrosis: molecular mechanisms and clinical implications. Thorax. (2013) 68(12):1157–62. 10.1136/thoraxjnl-2013-203204 23704228

[60] Bhatt L Roinestad K Van T Springman EB. Recent advances in clinical development of leukotriene B4 pathway drugs. Semin Immunol. (2017) 33:65–73. 10.1016/j.smim.2017.08.007 29042030

[61] Ramalho T Pereira N Brandt SL Serezani CH. Targeting leukotrienes as a therapeutic strategy to prevent comorbidities associated with metabolic stress. Adv Exp Med Biol. (2020) 1274:55–69. 10.1007/978-3-030-50621-6_4 32894507

[62] Galietta LJV. TMEM16A (ANO1) as a therapeutic target in cystic fibrosis. Curr Opin Pharmacol. (2022) 64:102206. 10.1016/j.coph.2022.102206 35364521

[63] Figueira MF Ribeiro CMP Button B. Mucus-targeting therapies of defective mucus clearance for cystic fibrosis: a short review. Curr Opin Pharmacol. (2022) 65:102248. 10.1016/j.coph.2022.102248) 35689870 PMC9891491

[64] Davies JC Sermet-Gaudelus I Naehrlich L Harris RS Campbell D Ahluwalia N A phase 3, double-blind, parallel-group study to evaluate the efficacy and safety of tezacaftor in combination with ivacaftor in participants 6 through 11 years of age with cystic fibrosis homozygous for F508del or heterozygous for the F508del-CFTR mutation and a residual function mutation. J Cyst Fibros. (2021) 20(1):68–77. 10.1016/j.jcf.2020.07.023 32967799

[65] Rowe SM McColley SA Rietschel E Li X Bell SC Konstan MW Effect of 8 weeks of lumacaftor in combination with ivacaftor in patients with CF and heterozygous for the F508del CFTR mutation. Pediatr Pulmonol. (2014) 49(Suppl 38):306.

[66] Wainwright CE Elborn JS Ramsey BW Marigowda G Huang X Cipolli M Lumacaftor-ivacaftor in patients with cystic fibrosis homozygous for Phe508del CFTR. N Engl J Med. (2015) 373(3):220–31. 10.1056/NEJMoa1409547 25981758 PMC4764353

[67] Rosenfeld M Marigowda G Liu F Waltz D. Effect of lumacaftor in combination with ivacaftor on FEV1 and safety measures in patients aged 6–11 years with CF who are homozygous for F508del-CFTR. Pediatr Pulmonol. (2014) 49(Suppl S38):287.

[68] Two 24-week Phase 3 Studies of Lumacaftor in Combination with Ivacaftor Met Primary Endpoint with Statistically Significant Improvements in Lung Function (FEV1) in People with Cystic Fibrosis who Have Two Copies of the F508del Mutation. USA: Vertex Pharmaceuticals Incorporated (2014). Available online at: http://investorsvrtxcom/releasedetailcfm?ReleaseID=856185

[69] Veit G Avramescu RG Perdomo D Phuan PW Bagdany M Apaja PM Some gating potentiators, including VX-770, diminish *Δ*F508-CFTR functional expression. Sci Transl Med. (2014) 6(246):246ra97. 10.1126/scitranslmed.3008889 25101887 PMC4467693

[70] Cholon DM Quinney NL Fulcher ML Esther CR Jr Das J Dokholyan NV Potentiator ivacaftor abrogates pharmacological correction of *Δ*F508 CFTR in cystic fibrosis. Sci Transl Med. (2014) 6(246):246ra96. 10.1126/scitranslmed.3008680 25101886 PMC4272825

[71] Liu X Dawson DC. Cystic fibrosis transmembrane conductance regulator (CFTR) potentiators protect G551D but not *Δ*F508 CFTR from thermal instability. Biochemistry. (2014) 53(35):5613–8. 10.1021/bi501007v 25148434 PMC4159205

[72] Pilewski J Donaldson S Cooke J Lekstrom-Himes J. Phase 2 studies reveal additive effects of VX-661, and investigational CFTR corrector, and ivacaftor, a CFTR potentiator, in patients with CF who carry the F508del-CFTR mutation. Pediatric Pulmonol. (2014) 49(Suppl 38):S157.

[73] Hutt DM Herman D Rodrigues AP Noel S Pilewski JM Matteson J Reduced histone deacetylase 7 activity restores function to misfolded CFTR in cystic fibrosis. Nat Chem Biol. (2010) 6(1):25–33. 10.1038/nchembio.275 19966789 PMC2901172

[74] Mu TW Ong DS Wang YJ Balch WE Yates JR 3rd Segatori L Chemical and biological approaches synergize to ameliorate protein-folding diseases. Cell. (2008) 134(5):769–81. 10.1016/j.cell.2008.06.037 18775310 PMC2650088

[75] Varga K Goldstein RF Jurkuvenaite A Chen L Matalon S Sorscher EJ Enhanced cell-surface stability of rescued DeltaF508 cystic fibrosis transmembrane conductance regulator (CFTR) by pharmacological chaperones. Biochem J. (2008) 410(3):555–64. 10.1042/BJ20071420 18052931 PMC3939615

[76] Young A Gentzsch M Abban CY Jia Y Meneses PI Bridges RJ Dynasore inhibits removal of wild-type and DeltaF508 cystic fibrosis transmembrane conductance regulator (CFTR) from the plasma membrane. Biochem J. (2009) 421(3):377–85. 10.1042/BJ20090389 19442237

[77] Allen L Allen L Carr SB Davies G Downey D Egan M Future therapies for cystic fibrosis. Nat Commun. (2023) 14(1):693. 10.1038/s41467-023-36244-2 36755044 PMC9907205

[78] Oren YS Sinai MIT Golec A Barchad-Avitzur O Mutyam V Li Y Antisense oligonucleotide-based drug development for cystic fibrosis patients carrying the 3849+10kb C-to-T splicing mutation. J Cyst Fibros*.* 20, 865–75 (2021). 10.1016/j.jcf.2021.06.003 34226157 PMC8464507

